# Management of acute pulmonary embolism 2019: what is new in the updated European guidelines?

**DOI:** 10.1007/s11739-020-02340-0

**Published:** 2020-05-26

**Authors:** Stavros Konstantinides, Guy Meyer

**Affiliations:** 1 Center for Thrombosis and Hemostasis (CTH), University Medical Center Mainz, Langenbeckstrasse 1, Bldg. 403, 55131 Mainz, Germany; 2 Department of Cardiology, Democritus University of Thrace, Komotini, Greece; 3 Respiratory Medicine Department, Hôpital Européen Georges Pompidou, APHP, Paris, France; 4 Université Paris Descartes, 75006 Paris, France

**Keywords:** Pulmonary embolism, Diagnosis, Treatment, Anticoagulation, Guidelines

## Abstract

Pulmonary embolism (PE) is the third most frequent acute cardiovascular syndrome. Annual PE incidence and PE-related mortality rates rise exponentially with age, and consequently, the disease burden imposed by PE on the society continues to rise as the population ages worldwide. Recently published landmark trials provided the basis for new or changed recommendations included in the 2019 update of the European Society of Cardiology Guidelines (developed in cooperation with the European Respiratory Society). Refinements in diagnostic algorithms were proposed and validated, increasing the specificity of pre-test clinical probability and d-dimer testing, and thus helping to avoid unnecessary pulmonary angiograms. Improved diagnostic strategies were also successfully tested in pregnant women with suspected PE. Non-vitamin K antagonist oral anticoagulants (NOACs) are now the preferred agents for treating the majority of patients with PE, both in the acute phase (with or without a brief lead-in period of parenteral heparin or fondaparinux) and over the long term. Primary reperfusion is reserved for haemodynamically unstable patients. Besides, the 2019 Guidelines endorse multidisciplinary teams for coordinating the acute-phase management of high-risk and (in selected cases) intermediate-risk PE. For normotensive patients, physicians are advised to include the assessment of the right ventricle on top of clinical severity scores in further risk stratification, especially if early discharge of the patient is envisaged. Further important updates include guidance (1) on extended anticoagulation after PE, taking into account the improved safety profile of NOACs; and (2) on the overall care and follow-up of patients who have suffered PE, with the aim to prevent, detect and treat late sequelae of venous thromboembolism.

## Acute pulmonary embolism: magnitude of the problem

Venous thromboembolism (VTE), clinically presenting as deep vein thrombosis (DVT) or acute pulmonary embolism (PE), is the third most frequent acute cardiovascular syndrome after myocardial infarction and stroke [1]. Annual incidence rates for PE lie between 39 and 115 per 100,000 population; for DVT, the rates are 53–162 per 100,000 [2]. The incidence of VTE is almost eight times higher in individuals aged 80 years or older than in the fifth decade of life [2]; consequently, and as society’s age, longitudinal studies keep showing a rising tendency in annual PE incidence rates [3–6] over time.

A recent analysis of vital registration data from the World Health Organisation (WHO) Mortality Database (2000–2015) reported an average of 38,929 PE-related deaths each year in 41 states of the WHO European Region (which includes Central Asia) with a total population of approximately 651 million [7]. Between 2000 and 2015, annual age-standardised PE-related mortality rates decreased by almost 50% (from 12.7 to 6.5 deaths per 100,000 population) without substantial sex-specific differences. Despite this overall favourable trend, the study also showed (1) that PE-related mortality continues to rise exponentially with age, reaching or even exceeding 80 deaths per 100,000 population among the elderly; and (2) that PE also remains a relatively important cause (compared to other causes) of death among younger women, in whom it accounted for up to 13 cases per 1000 deaths [7].

The PE Guidelines of the European Society of Cardiology (ESC), developed in cooperation with the European Respiratory Society (ERS), were updated in 2019 [8]. This article reviews the most important new or changed recommendations along with the recent evidence that provided their rationale and has begun to change clinical practice in several aspects of PE management.

## Refinements in established diagnostic algorithms, and extension of their use to suspected pulmonary embolism in pregnancy

Although the diagnostic steps in the proposed algorithms for the work-up of suspected acute PE have largely remained unchanged since the 2008 ESC Guidelines, refinements continue to be made to increase the specificity of pre-test clinical probability and d-dimer testing with the aim of limiting unnecessary computed tomography pulmonary angiography (CTPA). In particular, the following approaches were successfully validated in recent multicentre management trials:

- Since the specificity of d-dimer testing in suspected PE decreases steadily with age [9], a multinational prospective cohort study evaluated a previously developed age-adjusted cut-off (age × 10 µg/L for patients older than 50 years) in a cohort of 3346 patients [10]. Patients with a normal age-adjusted d-dimer value did not undergo CTPA, but were left without anticoagulation and followed for a 3-month period. In patients aged > 75 years, using the age-adjusted (instead of the standard 500 µg/L) d-dimer cut-off increased the number of patients in whom PE could be excluded from 6.4% to 30%, without adding false-negative findings [10].
- Another prospective management trial used the so-called ‘YEARS’ clinical decision rule, which consists of three clinical items of the Wells score, namely signs of DVT, haemoptysis, and ‘PE more likely than an alternative diagnosis’, combined with d-dimer concentrations [11]. The diagnosis of PE was rejected without further testing in patients without clinical items and d-dimer levels < 1000 ng/mL, and in patients with at least one clinical item and d-dimers < 500 ng/mL. The remaining patients underwent CTPA. Of the 2946 patients (85%) in whom PE was thus ruled out and who were left untreated, 18 (0.61%, 95% confidence interval [CI] 0.36–0.96%) were diagnosed with symptomatic VTE during the 3-month follow-up [11]. Using the YEARS rule allowed to exclude PE without CTPA in 48% of the patients as compared to 34% if the original Wells’ rule and fixed d-dimer threshold of less than 500 ng/mL had been applied [11].

Based on these results, the guidelines advise to consider either age-adjusted d-dimer cut-offs or the YEARS model as an alternative to the standard interpretation of the d-dimer test with a fixed cut-off level, which also remains a valid option of course [8].

Diagnosis of PE during pregnancy can be challenging as symptoms frequently overlap with those of normal pregnancy. Furthermore, d-dimer levels continuously increase during pregnancy [12, 13] and it has been reported that levels can be above the 500 µg/L threshold in up to 25% of pregnant women in the third trimester [13]. Moreover, registry data suggested that d-dimer testing might also be of limited sensitivity in this setting [14]. Aiming to clarify the situation, a multinational prospective management trial included 441 pregnant women presenting to emergency departments with clinically suspected PE. The results showed that a diagnostic strategy based on the assessment of clinical probability, d-dimer measurement, compression ultrasound of the leg veins, and CTPA may safely exclude PE in pregnancy [15]. A further prospective management study evaluated a combination of a pregnancy-adapted YEARS algorithm with d-dimer levels in 498 women with suspected PE during pregnancy. At 3 months, only one woman with PE excluded on the basis of the algorithm developed a popliteal DVT (0.21%; 95% CI 0.04–1.2) and no woman developed PE [16]. These results add further support to the pre-existing guidelines’ recommendation to adhere to formal diagnostic assessment with validated methods if PE is suspected during pregnancy or in the postpartum period.

## Risk-adjusted anticoagulation strategies

In patients with high or intermediate clinical probability for PE, anticoagulation should be initiated already upon suspicion and while awaiting the results of diagnostic tests. Parenteral anticoagulation may consist of subcutaneous, weight-adjusted low-molecular-weight heparin (LMWH) or fondaparinux, or intravenous unfractionated heparin (UFH). However, in haemodynamically stable patients not necessitating thrombolytic, surgical or interventional treatment, anticoagulation can now also be started via the oral route right away, using one of the non-vitamin K antagonist oral anticoagulants (NOAC) apixaban or rivaroxaban. This is in view of phase III clinical trials which demonstrated the non-inferior efficacy and superior safety of a single-oral-drug anticoagulation strategy, using higher doses of apixaban over the first 7 days or rivaroxaban over the first 3 weeks; the comparator arm received the traditional regimen of LMWH over at least 5 days, overlapping with and followed by a vitamin K antagonist (VKA) [17, 18].

Regardless of whether parenteral heparin is used over the first few hours or days after acute PE, the 2019 Guidelines now recommend that, when it is decided to start oral anticoagulation, a NOAC should be preferred to a vitamin K antagonist (VKA) [8]. This strong (class I, A) recommendation is based on the evidence from large clinical trials which led to the approval of three direct coagulation factor Xa inhibitors (apixaban, edoxaban, and rivaroxaban) and one thrombin inhibitor (dabigatran) for VTE treatment, and on real-world experience with these drugs which has accumulated since the previous (2014) guidelines. Of note, NOACs should not be given to patients with severe renal impairment, during pregnancy and lactation, and in patients with the antiphospholipid antibody syndrome. The European Heart Rhythm Association has provided and regularly updates a practical guide for the use of NOACs in everyday practice, and for the management of emergency situations related to anticoagulation [19].

## Advanced treatment and support of the patient with high-risk and intermediate–high-risk PE

Table 1 shows the new, extended criteria of haemodynamic instability defining acute high-risk PE, which have been adapted to be in accordance with the criteria used by the other ESC guidelines, notably those on acute and chronic heart failure.

**Table 1:** Updated definition of haemodynamic instability related to acute pulmonary embolism

(1) Cardiac arrest	(2) Obstructive shock (based on [20–22])	(3) Persistent hypotension
Need for cardiopulmonary resuscitation	Systolic BP < 90 mmHg, or vasopressors required to achieve a BP ≥ 90 mmHg despite adequate filling status	Systolic BP < 90 mmHg, or systolic BP drop ≥ 40 mmHg, either lasting longer than 15 min and not caused by new-onset arrhythmia, hypovolaemia, or sepsis
	And	
	End-organ hypoperfusion (altered mental status; cold, clammy skin; oliguria/anuria; increased serum lactate)	

*BP* blood pressure

In suspected acute high-risk PE, the clinical probability is usually high, and the differential diagnosis includes other life-threatening situations such as cardiac tamponade, acute coronary syndrome, aortic dissection, acute valvular dysfunction, and hypovolaemia. Immediate bedside transthoracic echocardiography will detect acute RV dysfunction if acute PE is the cause of the patient’s haemodynamic decompensation. In a highly unstable patient, echocardiographic evidence of RV dysfunction is sufficient to prompt immediate reperfusion without further testing. In intubated patients, transoesophageal echocardiography may allow direct visualisation of thrombi in the pulmonary artery and its main branches, especially in patients with RV dysfunction.

The 2019 guidelines recommend to consider setting up a multidisciplinary team for acute-phase management of high-risk and (in selected cases) intermediate-risk PE, depending on the resources and expertise available in each hospital [8]. Primary reperfusion treatment, in most cases systemic thrombolysis, is the treatment of choice for patients with high-risk PE. Surgical pulmonary embolectomy or percutaneous catheter-directed treatment is alternative reperfusion options in patients with contraindications to thrombolysis, if expertise with either of these methods and the appropriate resources are available on site. Extracorporeal membrane oxygenation (ECMO) may be life-saving in patients with PE and refractory circulatory collapse or cardiac arrest, but only when used as a bridge to surgical embolectomy or catheter-directed treatment [23].

In contrast to the clinical setting of high-risk PE, routine use of primary systemic thrombolysis is not recommended in patients with intermediate-risk PE [24]. However, patients with intermediate–high-risk PE should be monitored, clinically and haemodynamically, and rescue thrombolytic therapy should be performed in case of overt or imminent haemodynamic decompensation on anticoagulation treatment [25]. As an alternative option, surgical embolectomy or percutaneous catheter-directed treatment should be considered in this latter situation [8].

## Definition of low-risk PE and implications for early discharge and home (outpatient) treatment

Principally, three sets of criteria need to be fulfilled before considering early discharge and home treatment of a patient with acute PE: (1) a low risk of early PE-related life-threatening or serious complications that would require a prolonged hospital stay or (re)hospitalisation; (2) absence of serious comorbidity or aggravating conditions; and (3) the certainty (as much as this is possible) of proper outpatient care and anticoagulant treatment, taking into account the patient’s anticipated compliance, his/her family and social environment, and a local infrastructure permitting rapid access to medical care if necessary.

The PESI, in its original and simplified form (sPESI), integrates clinical parameters of PE severity and comorbidity, and its ability to permit reliable assessment of overall 30-day mortality has been validated in multiple cohorts. Currently, it is the most frequently used tool for risk stratification of acute PE. However, the PESI was not primarily developed as a tool for selecting candidates for home treatment, and it is, therefore, needed to be combined with additional feasibility criteria in a trial which randomised 344 patients to inpatient versus outpatient treatment [26]. Moreover, the sPESI automatically classifies all patients above the age of 80 as well as those with cancer into an ‘elevated risk’ category, but it is not clear whether these patients should a priori be excluded from an early discharge management strategy in the absence of any other criteria of PE severity or serious comorbidity. As an alternative to the (s)PESI, the Hestia exclusion criteria represent a checklist of clinical parameters or questions that can be obtained/answered at the bedside. They integrate PE severity, comorbidity, and feasibility of home treatment. If the answer to one or more of the questions is ‘yes’, then the patient cannot be discharged early. Neither age nor the diagnosis of cancer is among the Hestia criteria. The Hestia criteria safely identified candidates for the early discharge in a single-arm management trial and in a subsequent non-inferiority study [27, 28].

In the context of the physician’s increased responsibility when deciding to send home a patient with an acute cardiovascular syndrome such as PE, the definition of ‘truly low-risk’ PE may require findings beyond those included in clinical scores. This requirement mainly refers to an assessment of RV size, function, and intracardiac thrombi by echocardiography or CTPA. A recent systematic review and meta-analysis of cohort studies supports the notion that the prognostic sensitivity can further be improved when the PESI or sPESI is combined with absence of RV dysfunction on imaging, or with normal laboratory biomarker (mostly troponin) levels [29]. Furthermore, a multinational prospective management trial investigated the efficacy and safety of early discharge and ambulatory rivaroxaban treatment in patients selected by clinical criteria and the absence of RV dysfunction. Overall, a little less than 20% of the screened unselected patients with PE were included. At the predefined interim analysis of 525 patients (50% of the planned population), the 3-month rate of symptomatic or fatal recurrent VTE was only 0.6%, approximately one-third of the initially estimated rate on the basis of previous studies, and this permitted the early termination of the trial. Major bleeding occurred in 6 (1.2%) of the patients in the safety population. There were no PE-related deaths [30].

In view of these data, the 2019 ESC/ERS guidelines recommend to include the assessment of the RV in the risk stratification of all patients with acute PE, on top of the PESI or sPESI score. In particular, the absence of RV dysfunction and right heart thrombi should be ensured before immediate or early (within 24–48 h) discharge of a patient with low-risk PE [8]. If bedside echocardiography, or a focused ultrasound examination of the heart, is not continuously available at the emergency department of a given hospital, local clinical protocols should request cardiac ultrasound if the CTPA assessment of the RV yields suspicious or inconclusive findings.

## An updated management algorithm for pulmonary embolism

**Fig. 1:**
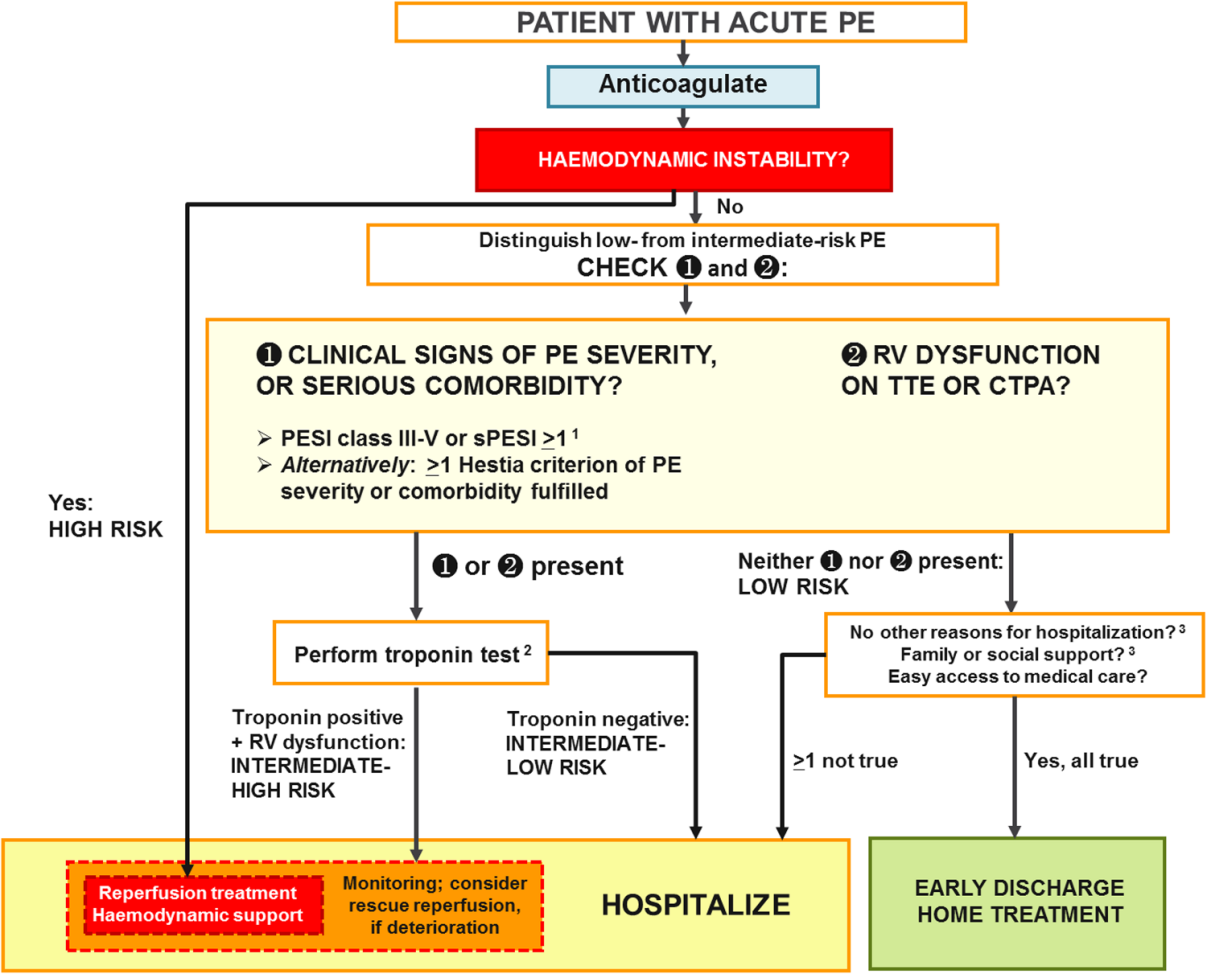
Risk-adjusted management strategies in acute PE ([8]). *CTPA* computed tomography pulmonary angiography/angiogram, *PE* pulmonary embolism, *PESI* Pulmonary Embolism Severity Index, *RV* right ventricular, *sPESI* simplified Pulmonary Embolism Severity Index, *TTE* transthoracic echocardiogram. ^1^Cancer, heart failure and chronic lung disease are the comorbidities included in the PESI and sPESI. ^2^A cardiac troponin test may already have been performed during the initial diagnostic work-up (e.g., in the chest pain unit). Troponin is proposed as the preferred biomarker, because it is the only one to have been used in an interventional trial [24]. ^3^Included in the Hestia criteria adapted from the 2019 European Society of Cardiology Guidelines

Based on the considerations explained above, the comprehensive risk-adapted management algorithm for acute PE was updated in the 2019 PE guidelines. It is shown in Fig. 1.

## Long-term and extended anticoagulation for secondary VTE prevention

Experts agree that, after the acute phase, all patients with PE should be treated with anticoagulants for at least 3 months [31]. At this time, therapeutic oral anticoagulation should be discontinued in patients who had a first PE event due to a major transient/reversible risk factor for thrombosis [8, 32–34]. Such risk factors include (1) surgery with general anaesthesia for > 30 min; (2) immobilisation in bed for ≥ 3 days due to an acute illness or acute exacerbation of a chronic illness; or (3) trauma with fractures. The recommendation for limiting anticoagulation to 3 months is based on the fact that the risk for late VTE recurrence is very low in these situations.

In contrast, the upper end of the risk spectrum with regard to late recurrence includes patients with (1) recurrent VTE, i.e., with at least one previous episode of PE or DVT [35]; (2) the antiphospholipid syndrome; and (3) cancer, at least until the cancer is considered ‘cured’. In these cases, continuation of oral anticoagulant treatment is recommended indefinitely.

In all other clinical settings, including (1) the presence of a minor transient or reversible risk factor for thrombosis (e.g., long-haul travel); (2) a persistent risk factor other than the antiphospholipid syndrome (e.g., chronic inflammatory disease); or (3) the absence of any identifiable risk factor, the most reasonable approach is to base the decision regarding extension of anticoagulant treatment on a personalised balance between recurrence versus bleeding risk. It should be noted that this balance is currently shifting in favour of indefinitely extending treatment in the majority of cases, in view of the lower bleeding rates with NOACs (compared to historical studies using VKA) and the favourable results of extension trials with these drugs. Consequently, the 2019 guidelines recommend to consider continuation of treatment in all patients belonging to this category [8], meaning that the decision process should be explicit and the rationale for continuing or not should be documented in the patient’s record. In this context, bleeding risk assessment, either by clinical evaluation of individual risk factors or by the use of an integrated bleeding risk score, principally serves the aim of identifying and treating modifiable bleeding risk factors, but it may also influence the duration and dose of anticoagulant treatment after PE.

If extended oral anticoagulation is decided after PE, a reduced dose of the NOACs apixaban (2.5 mg b.i.d.) or rivaroxaban (10 mg o.d.) should be considered after 6 months of therapeutic anticoagulation [36, 37]. Patients with the antiphospholipid syndrome should generally be treated with a VKA, especially if they test triple positive for lupus anticoagulant, anticardiolipin antibodies, and beta2-glycoprotein I antibodies [38, 39].

Patients with PE and active cancer belong to a separate risk category, with a higher frequency of both VTE recurrence and bleeding on standard anticoagulation compared to non-cancer patients. A prolonged initial treatment period with weight-adjusted subcutaneous LMWH is currently recommended by most experts in view of the documented superior efficacy of LMWH compared to VKA. LMWH can be switched to oral anticoagulation after the first 3–6 months. More recently, however, two dedicated trials provided data showing (at least) non-inferior efficacy of edoxaban, and in a smaller trial rivaroxaban, in patients with cancer [40, 41]. Consequently, the 2019 guidelines recommend to consider these drugs as an alternative to LMWH in this setting [8]; this statement is accompanied by a word of caution for patients with gastrointestinal cancer due to the increased bleeding risk reported with these NOACs in both trials [40, 41]. The ESC guideline recommendations are thus largely in agreement with a consensus statement recently issued by the International Society on Thrombosis and Haemostasis [42]. The results of a further large trial comparing apixaban with LMWH for treatment of VTE in patients with cancer are expected soon [43].

The 2019 guidelines also provide advice for the anticoagulation management of PE in specific clinical situations, for which conclusive evidence is lacking to this date (Table 2) [8].

**Table 2:** Guidance for management of pulmonary embolism in ‘borderline’ clinical situations, for which no solid evidence exists ([8]) adapted from the 2019 European Society of Cardiology Guidelines

Clinical setting	Suggested management	Comments
Subsegmental PE	Single subsegmental PE in an outpatient without cancer and without proximal DVT: Clinical surveillance Single subsegmental PE in a hospitalised patient, or a patient with cancer, or if associated with confirmed proximal DVT: Anticoagulant treatment Multiple subsegmental PE: Anticoagulant treatment	Poor interobserver agreement for the diagnosis of subsegmental PE; diagnosis to be confirmed by an experienced thoracic radiologist Suggestion based on indirect evidence, only limited data available
Incidental PE	If single subsegmental PE: Proceed as above In all other cases: Αnticoagulant treatment	Suggestion based on retrospective cohort data
Management of acute PE in a patient with active bleeding	Insert inferior vena cava filter (preferably retrievable) Reassess the possibility of anticoagulation as soon as the bleeding has ceased and the patient is stabilised, and remove the filter as soon as anticoagulant treatment is resumed	
PE diagnosis and anticoagulation in the elderly, frail patient, and the patient with polypharmacy	Assess clinical probability of PE as in the non-frail patient, but caution needed in the nursing home setting as clinical prediction rules may be unreliable [44] Generally prefer NOACs over VKAs in elderly and frail patients, but observe the following: (a) Avoid NOACs in patients with severe renal impairment (b) Consult the drugs’ SPC and the updated EHRA guide [19] for possible interactions between NOACs and the patient’s concomitant medication Reassess, at regular intervals, drug tolerance and adherence, hepatic and renal function, and the patient’s bleeding risk	Number of diseases mimicking PE symptoms increases with age, making diagnostic delay more common These patients were poorly represented in clinical trials. Whatever the treatment (VKAs or NOACs), these patients are at high risk of bleeding
Management of acute PE in a patient with signs of chronic pulmonary hypertension on TTE, or findings suggesting pre-existing CTEPH on CTPA (suspected ‘acute-on-chronic’ PE)	If the diagnosis of acute PE has been confirmed, focus on the patient’s acute problem and proceed to risk-adjusted acute-phase treatment of PE as described in Fig. 1 Perform a TTE upon discharge and document any signs of persisting pulmonary hypertension or RV dysfunction Continue anticoagulation for at least 3 months and schedule the patient for a 3-month follow-up visit At the 3-month follow-up visit, assess the presence of persisting or worsening symptoms, or functional limitation, perform follow-up TTE and consider further tests before possible referral to a PH/CTEPH expert centre	
Initial anticoagulation in the patient with acute PE and end-stage renal disease	Administer UFH; consider anti-Xa (rather than aPTT) monitoring [45]	No truly safe anticoagulation option available, although LMWH with anti-Xa monitoring is also used in clinical practice
Duration of anticoagulation in the young female patient suffering acute PE while on oral contraceptives	If patient was taking an oestrogen-containing contraceptive, and especially if PE occurred in the first 3 months of initiation of contraception: Discontinue hormonal contraceptives after discussing alternative methods of contraception; consider discontinuing anticoagulation after 3 months All other cases: Manage chronic anticoagulation as after acute PE occurring in the absence of identifiable risk factors Consider using a validated prediction model for quantification of the risk for VTE recurrence; for example, the HERDOO2 score: (a) hyperpigmentation, oedema, or redness in either leg; (b) d-dimer level ≥ 250 μg/L; (c) obesity with body mass index ≥ 30; (d) older age (essentially 0 in this case) A score of 0 or 1 may help identify young women who can safely discontinue anticoagulant treatment Advise patient on the need for prophylaxis with LMWH in case of pregnancy	The risk of VTE attributable to oestrogen–progestin contraception (or hormonal treatment) depends on the specific compound and the presence of concomitant thrombophilia, and is associated with the time interval between initiation of hormonal treatment and the occurrence of acute PE [46, 47]
Anticoagulation in the patient with PE and cancer, after the first 6 months	If cancer still active^a^ Continue anticoagulation with LMWH or, alternatively, edoxaban or rivaroxaban If cancer in remission: Continue oral anticoagulation (NOAC or VKA); alternatively, consider discontinuing if the bleeding risk is high. In either case, periodically reassess the risk–benefit ratio of continuing/resuming anticoagulation	In the absence of conclusive evidence, the decision to continue or stop after the first 6 months anticoagulation should be made on a case-by-case basis after considering the success of anti-cancer therapy, the estimated overall risk of VTE recurrence, the bleeding risk, and the preference of the patient

*aPTT* activated partial thromboplastin time, *CrCl* creatinine clearance, *CTEPH* chronic thromboembolic pulmonary hypertension, *CTPA* computed tomography pulmonary angiography, *DVT* deep vein thrombosis, *EHRA* European Heart Rhythm Association, *HERDOO2* Hyperpigmentation, Edema, or Redness in either leg; d-dimer level ≥ 250 μg/L; obesity with body mass index ≥ 30; or older age, ≥ 65 years, *LMWH* low-molecular-weight heparin, *NOAC(s)* non-vitamin K antagonist oral anticoagulant(s), *PE* pulmonary embolism, *PH* pulmonary hypertension, *RV* right ventricular, *SPC* summary of product characteristics, *TTE* transthoracic echocardiography/echocardiogram, *UFH* unfractionated heparin, *VKA(s)* vitamin K antagonist(s), *VTE* venous thromboembolism

^a^Recurrent, regionally advanced, or metastatic cancer; cancer for which treatment has been administered in the past 6 months; or haematologic cancer that is not in complete remission.

## Take-home messages for the contemporary management of acute PE

The changes and updates included in the 2019 European Guidelines can be summarised into ‘ten commandments’ [48], which may help the clinicians to improve the management of suspected or confirmed PE in their practice:

1. Perform bedside transthoracic echocardiography as the immediate diagnostic test of choice in a patient presenting with haemodynamic instability (suspected high-risk PE).
2. For haemodynamically stable patients with suspected PE, use a validated diagnostic algorithm including a standardised assessment of pre-test clinical probability and d-dimer testing.
3. Start anticoagulation therapy upon suspicion acute PE, while the diagnostic work-up is ongoing, if the clinical (pre-test) probability is intermediate or high, unless there is active bleeding or the patient has absolute contraindications to anticoagulants.
4. If the CTPA report speaks of single subsegmental PE, discuss the findings again with the radiologist or obtain a second opinion to avoid misdiagnosis.
5. Evaluate the size and/or function of the RV along with clinical findings and comorbidity in all patients presenting without haemodynamic instability.
6. In a patient with strongly suspected or confirmed high-risk PE, and in initially normotensive patients with haemodynamic decompensation after admission to the hospital, determine the best reperfusion option (systemic thrombolysis, surgical embolectomy, or catheter-directed treatment), through consensus in an interdisciplinary team, taking into account the resources and expertise available at your hospital.
7. Start oral anticoagulation with an NOAC, as these drugs have become the standard of care for the majority of patients with acute PE. The LMWH − VKA regimen is an alternative for patients with contraindications to NOACs.
8. In a patient who suffered acute PE not provoked by a strong transient/reversible risk factor, perform a personalised assessment of the benefits versus risks of continuing anticoagulation treatment after the first 3–6 months. Consider the good safety profile of NOACs in your decision, and also take into account the patient’s fears and preferences. Do not forget to perform regular follow-up examinations, as a rule once a year.
9. If PE is suspected in a pregnant patient, use formal diagnostic pathways and risk assessment. If needed, do not hesitate to perform a CTPA or ventilation–perfusion lung scan to reliably confirm or exclude the diagnosis. Manage haemodynamically unstable pregnant patients based on the same emergency algorithm as for non-pregnant patients.
10. Follow the patient after acute PE at regular intervals. Check for possible signs of VTE recurrence, cancer, or bleeding complications of anticoagulants. In addition, if the patient reports persisting or new-onset dyspnoea or functional limitation, of if there are predisposing conditions for chronic thromboembolic pulmonary hypertension (CTEPH), evaluate the RV by echocardiography combined with natriuretic peptide measurements and possibly cardiopulmonary exercise testing. If this initial work-up generates the suspicion of CTEPH or chronic thromboembolic disease, refer the patient to an expert pulmonary hypertension/CTEPH centre.
